# EANM guidance document: dosimetry for first-in-human studies and early phase clinical trials

**DOI:** 10.1007/s00259-024-06640-x

**Published:** 2024-02-17

**Authors:** Caroline Stokke, Silvano Gnesin, Johannes Tran-Gia, Francesco Cicone, Søren Holm, Marta Cremonesi, Johan Blakkisrud, Thomas Wendler, Nic Gillings, Ken Herrmann, Felix M. Mottaghy, Jonathan Gear

**Affiliations:** 1 Department of Diagnostic Physics and Computational Radiology, Division of Radiology and Nuclear Medicine, Oslo University Hospital, Oslo, Norway; 2 Department of Physics, University of Oslo, Oslo, Norway; 3 Institute of Radiation Physics, Lausanne University Hospital and University of Lausanne, Lausanne, Switzerland; 4 Department of Nuclear Medicine, University Hospital Würzburg, Würzburg, Germany; 5 Nuclear Medicine Unit, Department of Experimental and Clinical Medicine, “Magna Graecia” University of Catanzaro, Catanzaro, Italy; 6 Department of Clinical Physiology and Nuclear Medicine, Copenhagen University Hospital Rigshospitalet, Copenhagen, Denmark; 7 Department of Medical Imaging and Radiation Sciences, European Institute of Oncology, IRCCS, Milan, Italy; 8 Computer-Aided Medical Procedures and Augmented Reality, Technische Universität München, Munich, Germany; 9 Clinical Computational Medical Imaging Research, Department of Diagnostic and Interventional Radiology and Neuroradiology, University Hospital Augsburg, Augsburg, Germany; 10 Department of Nuclear Medicine, University of Duisburg-Essen, and German Cancer Consortium (DKTK)-University Hospital Essen, Essen, Germany; 11 National Center for Tumor Diseases (NCT), NCT West, Heidelberg, Germany; 12 Department of Radiology and Nuclear Medicine, GROW - School for Oncology and Developmental Biology, Maastricht University Medical Centre, Maastricht, The Netherlands; 13 Department of Nuclear Medicine, University Hospital RWTH Aachen, Aachen, Germany; 14 Joint Department of Physics, Royal Marsden NHSFT & Institute of Cancer Research, Sutton, UK

**Keywords:** European Association of Nuclear Medicine, Dosimetry, First-in-human, Nuclear medicine, Radiopharmaceuticals

## Abstract

The numbers of diagnostic and therapeutic nuclear medicine agents under investigation are rapidly increasing. Both novel emitters and novel carrier molecules require careful selection of measurement procedures. This document provides guidance relevant to dosimetry for first-in human and early phase clinical trials of such novel agents. The guideline includes a short introduction to different emitters and carrier molecules, followed by recommendations on the methods for activity measurement, pharmacokinetic analyses, as well as absorbed dose calculations and uncertainty analyses. The optimal use of preclinical information and studies involving diagnostic analogues is discussed. Good practice reporting is emphasised, and relevant dosimetry parameters and method descriptions to be included are listed. Three examples of first-in-human dosimetry studies, both for diagnostic tracers and radionuclide therapies, are given.

**Supplementary Information:**

The online version contains supplementary material available at 10.1007/s00259-024-06640-x.

## Preamble

The European Association of Nuclear Medicine (EANM) is a professional nonprofit medical association that facilitates communication worldwide among individuals pursuing clinical and research excellence in nuclear medicine. The EANM was founded in 1985. These guidelines are intended to assist practitioners in providing appropriate nuclear medicine care for patients. They are not inflexible rules or requirements of practice and are not intended, nor should they be used, to establish a legal standard of care. The ultimate judgement regarding the propriety of any specific procedure or course of action must be made by medical professionals taking into account the unique circumstances of each case. Thus, there is no implication that an approach differing from the guidelines, standing alone, is below the standard of care. On the contrary, a conscientious practitioner may responsibly adopt a course of action different from that set out in the guidelines when, in the reasonable judgement of the practitioner, such course of action is indicated by the condition of the patient, limitations of available resources, or advances in knowledge or technology subsequent to the publication of the guidelines. The practice of medicine involves not only the science but also the art of dealing with the prevention, diagnosis, alleviation, and treatment of disease. The variety and complexity of human conditions make it impossible to always reach the most appropriate diagnosis or to predict with certainty a particular response to treatment. Therefore, it should be recognised that adherence to these guidelines will not ensure an accurate diagnosis or a successful outcome. All that should be expected is that the practitioner will follow a reasonable course of action based on current knowledge, available resources, and the needs of the patient to deliver effective and safe medical care. The sole purpose of these guidelines is to assist practitioners in achieving this objective.

## Introduction

**Fig. 1:**
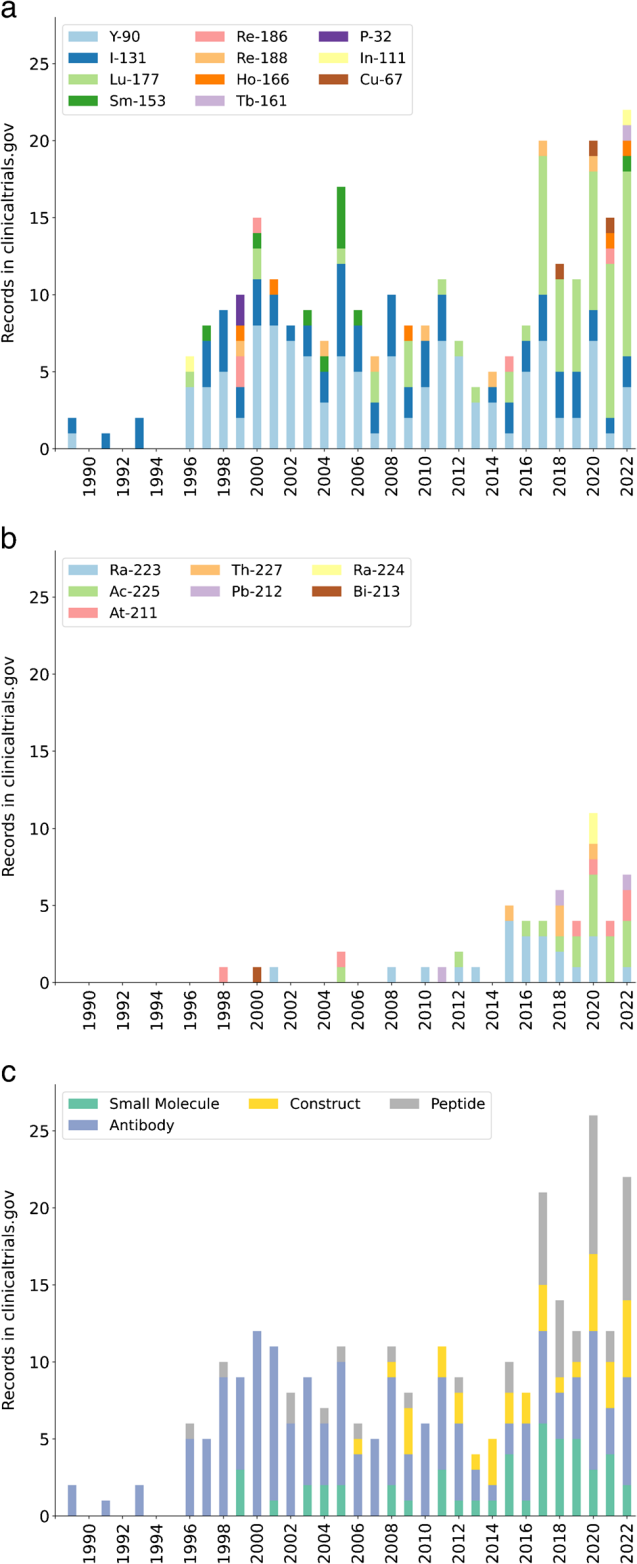
The number of phase 1 or early phase 1 interventional studies with therapeutic radionuclides and molecular or functional targeting mechanisms recorded in ClinicalTrials.gov per year, as of end of 2022. Electron emitters and alpha emitters are split in (**a**) and (**b**), and in (**c**), the results have been separated according to carrier instead of emitter

**Fig. 2:**
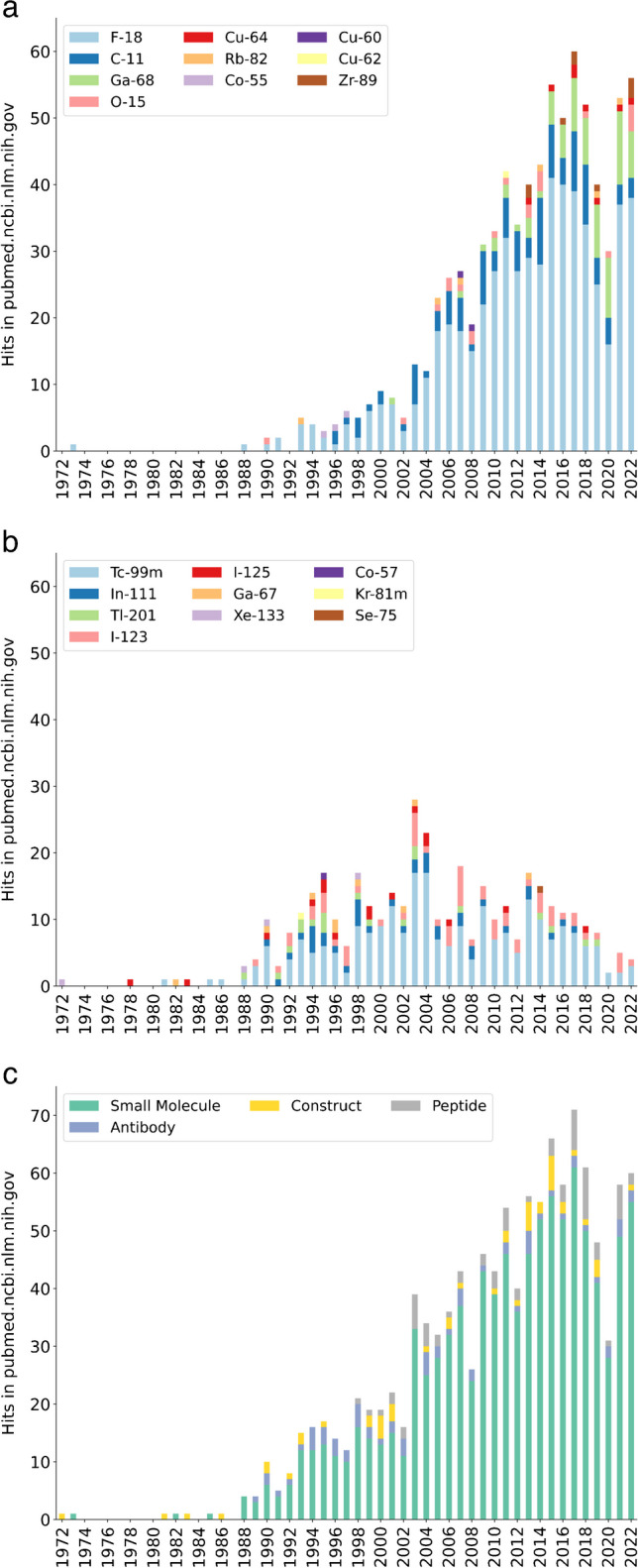
The number of publications with the Mesh terms “Pilot Projects” and “Radionuclide Imaging” and mention of the individual radionuclides in the title or abstract, as of end of 2022. The 1252 nuclides from ICRP 107 were included in the search, and radionuclides used unambiguously for therapy have been removed. **a** Emitters primarily used for PET imaging and **b** single photon emitters. **c** The hits separated by carrier

Over the last decade, up to 30 therapeutic nuclear medicine agents have been investigated per year in first-in-human or phase 1 studies (Fig. 1), and up to 70 articles per year describing pilot studies of radionuclide imaging have been published (Fig. 2). While these data will not represent the total number of first-in-human or early phase studies conducted, the overarching trends are clearly recognisable. Official bodies such as the European Medicines Agency (EMA) or the USA’s Food and Drug Administration (FDA) require that dosimetric evaluations are performed in order to receive marketing authorisation of new drugs. For example, EMA specifically mentioned radiation dosimetry in previous versions of their “Guideline on Radiopharmaceuticals/eudralex 3AQ20a” [1] where it was recommended that calculations of absorbed dose to organs should be carried out in accordance with the Medical Internal Radiation Dosimetry (MIRD) schemes, and the effective dose should be calculated using the current weighting factors established by the International Commission on Radiological Protection (ICRP). This was omitted in the 2008 version [1], after dosimetry and other clinical aspects were included in the European Directive 2001/83/EC [2]. In article 11 of the directive, it is stated that the product characteristics shall contain “full details of internal radiation dosimetry”, and furthermore in part III, under radio-pharmaceuticals; “Organ/tissue exposure to radiation shall be documented. Absorbed radiation dose estimates shall be calculated according to a specified, internationally recognised system by a particular route of administration” [2]. The FDA requires that “Phase 1 studies of radioactive drugs must include studies which will obtain sufficient data for dosimetry calculations” (title 21 CFR 312.23(a)(10)(ii), [3]), and for the labelling of products; “*Radiation dosimetry information must be stated for both the patient receiving a radioactive drug and the person administering it*” (title 21 CFR 201.57(c)(3)(iii), [3]). Also for diagnostic radiopharmaceuticals, safety evaluations include absorbed radiation doses (title 21 CFR 315.6 (d), [3]). While dosimetry is hence clearly required at an early stage of drug development, there are currently no detailed requirements or guidance provided on how to carry out the necessary investigations. Previous EANM guidelines have addressed good practice reporting and uncertainty analyses [4, 5], which are relevant for all forms of clinical dosimetry; otherwise, only guidance documents for specific treatments or targets exist.

First-in-human studies may include a wide range of emitters, carrier molecules, activity amounts, and administration routes, which can pose various challenges for dosimetric evaluations. While novel emitters require careful set-up and calibration of measurement instruments, novel carrier molecules may require just as careful selection of measurement time-points and procedures. In general, diagnostic tracers will most often allow for imaging approaches, while therapeutic agents—especially alpha-emitters—may require alternative methods. Information obtained from other agents with similar or identical carrier molecule can be used to guide the acquisition protocol, or directly inform the dosimetry. Information from animal studies will often also be present. The FDA requires that Investigational New Drug applications (INDs) contain “sufficient data from animal or human studies to allow a reasonable calculation of radiation-absorbed dose to the whole body and critical organs upon administration to a human subject” (title 21 CFR 312.23(a)(10)(ii)), [3]). A recent abstract from the FDA’s Center for Drug Evaluation and Research compared the ratios between human dose estimates derived from animals and from human measurements for carbon-11 and fluorine-18 imaging agents and found coefficients of variation of up to 60% for individual organs [6]. Large differences were also found in another study of two tracers: one peptidic and one based on an antibody fragment [7]. Although dosimetric translations from animals to humans should be performed with care, the animal biokinetics may provide relevant information. Early pharmacokinetic, biodistribution and dosimetry studies inform the data presented within the Summary of Product Characteristics. These data are then used by regulatory agencies and practitioners when evaluating a product for clinical use. The robustness and appropriateness of such data must therefore be maximised.

## Background information

### Radionuclides

Relevant types of emitters, including examples of currently common radionuclides, are listed in Table 1. Possible imaging methods, obtained from previous studies with focus on quantification and optimisation, are also provided. Imaging approaches are relatively straightforward for many nuclides, such as fluorine-18, technetium-99 m, and lutetium-177. However, some nuclides are more challenging. Imaging of alpha-emitters is often difficult due to the lower amounts of activity commonly administered, and pure beta-emitters such as yttrium-90 lack the primary photon emission to isolate for energy discrimination. The biodistribution, amount of activity, and equipment performance (e.g. energy range, sensitivity) may then determine whether imaging approaches are feasible or should be replaced or supplemented by other methods. Potential radioactive daughters, metastable states (e.g. lutetium-177 m), and other production impurity isotopes (e.g. rhenium-188 impurities in rhenium-186) should also be considered. While these are not included explicitly in Table 1, some of the photons listed are emitted by daughters. When imaging daughter emissions, care should be taken to ensure the imaging adequately indicates the position of the parent radionuclide rather than the biodistribution of different radionuclides in the decay chain. In addition, depending on the half-life and probability for relocalisation, separate dosimetry of the other radionuclides may need independent investigation.

**Table 1:** List of example radionuclides with principal emissions and imaging techniques

Principal emitted radiation	Examples of nuclides	Physical half-life	Selected photons emitted (yield)* and suggested imaging method	Example references related to imaging
Alpha	Radium-223	11.4 d	81–95 keV (52%), 144–154 keV (9%), 270–271 keV (25%)*, Gamma camera/SPECT	[8–10]
	Actinium-225	10.0 d	218 keV (12%)*, 440 keV (26%)*, Gamma camera/SPECT	[8, 11]
	Lead-212	10.6 h	239 keV (43%), 73–88 keV (approx. 40%), Gamma camera/SPECT	[12]
	Astatine-211	7.2 h	77–92 keV (45%)*, Gamma camera/SPECT	[13, 14]
Beta-	Iodine-131	8.0 d	364 keV (82%), SPECT	[15, 16]
	Lutetium-177	6.6 d	113 keV (6%), 208 keV (11%), SPECT	[17–19]
	Yttrium-90	64.1 h	511 keV (0,003%)*, PET (or bremsstrahlung SPECT)	[20–23]
	Copper-67	61.8 h	185 keV (49%), SPECT	[24]
	Holmium-166	23.8 h	81 keV (7%), SPECT or MRI	[25]
	Rhenium-186	3.7 d	137 keV (9%), SPECT	[26]
	Rhenium-188	17.0 h	155 keV (15%), SPECT	[26]
Beta-/auger	Terbium-161	6.9 d	75 keV (10%), SPECT	[27]
Gamma/auger	Iodine-123	13.2 h	159 keV (83%), SPECT	[28, 29]
	Indium-111	2.8 d	171 keV (91%), 245 keV (94%), SPECT	[30, 31]
	Technetium-99 m	6.0 h	Approx. 141 keV (100%), SPECT	[28]
Beta +	Fluorine-18	110 min	511 keV (97%), PET	[32]
	Carbon-11	20 min	511 keV (100%), PET	
	Gallium-68	68 min	511 keV (89%), PET	[33, 34]
	Copper-64	12.7 h	511 keV (18%)*, PET	[35]
	Zirconium-89	3.3 d	511 keV (23%), PET	[35, 36]

*Also include radioactive daughters

### Carrier molecules

A range of potential carriers exist, including antibodies and related structures (such as antibody fragments), peptides and proteins, cells, vehicles for non-targeted transport, and various small molecules. Radionuclides can also be injected in salt forms (e.g. [^131^I]NaI or [^89^Sr]SrCl_2_). In Table 2, examples of different carrier molecules are given, together with a few key pharmacokinetic features. While some overall features are demonstrated, such as prolonged circulation of antibodies compared to peptides or small molecules, the behaviour of individual compounds may vary greatly, and the normal tissues of interest and expected kinetics should be evaluated for each case. It should also be noted that the parameters in Table 2 are derived from studies of radiopharmaceuticals; therefore, pure carrier molecules, or carrier molecules labelled with other radionuclides, might not display identical behaviour. Non-targeted compounds are often special in the sense of being injected loco-regionally, and there is limited transference value for similar compounds unless they also share administration site.

**Table 2:** List of example carrier molecules derived from studies of radiopharmaceuticals

Class of carrier	Example carrier molecule	Example of disease or target	Biological WB and/or blood half-life	Normal tissues with uptake/involved in clearance	References
Antibodies	Ibritumomab	Non-Hodgkin lymphoma/CD20	Approx. 400 h (WB), 46 h (whole blood)	Spleen, liver, red marrow	[37, 38]
	Trastuzumab	Breast cancer/HER2	Approx. 400 h (WB), 11 and 176 h (blood; fast and slow)	Liver, kidneys	[39, 40]
Peptides	Somatostatin analogues	Neuroendocrine disease/somatostatin receptor	Approx. 1.3 and 71 h (WB; fast and slow), 1.6 and 59 h (blood; fast and slow)	Kidneys	[41]
	Prostate-specific membrane antigen (PSMA) targeting ligands	Metastatic castration-resistant prostate cancer/PSMA	Approx. 45–57 h (WB), 0.2 and 12 h (blood; fast and slow)	Lacrimal and salivary glands, kidneys	[42, 43]
Small molecules	Fluoro-2-deoxy-D-glucose (FDG)	Glucose metabolism	∞ or 12 min–1.5 h (WB), < 1 min, but also minor fraction with longer half-life (blood)	Kidneys and urinary tract, brain, heart	[44]
	(Fluoroethyl)-L-tyrosine (FET)	Amino acid metabolism	14 h (WB), < 0.05 h and 14 h (blood; fast and slow)	Liver, kidneys	[44]
	Fibroblast activation protein inhibitors	Tumour microenvironment	24–65 h (WB), 9 h (blood)	Kidneys and urinary tract	[45, 46]
	Meta-iodobenzylguanidine (mIBG)	Neuroendocrine disease/neurosecretory granules	9–130 h (blood)	Liver, adrenal glands, bone marrow	[47]
Non-targeted compounds	Microspheres for selective internal radiotherapy*	Disease in the liver	∞ (WB), N.A. (blood)	Local tissues	[20]

*Injected intra-arterially

### Administration route

Administrations can be intravenous, oral, inhalation-based, or performed by loco-regional injection (e.g. intra-arterial, intra-tumoural, or in cavities) or surface application (skin, on cavity surfaces). For oral, inhalation-based, or loco-regional injections and surface applications, stomach and gastrointestinal systems, lungs, and administration site should be included as potential source organs (see section “Biodistribution and time-integrated activity estimation” for definition of source organs).

## Relevant prior information

### Overview

Dosimetry investigations are among the preclinical safety data recommended in the EMA document “ICH guideline M3(R2) on non-clinical safety studies for the conduct of human clinical trials and marketing authorisation for pharmaceuticals” to support exploratory clinical trials [48]. The EMA “Guideline on the non-clinical requirements for radiopharmaceuticals” defines the requirements to be met by a new radiopharmaceutical in order to be translated clinically, and according to the 2018 version a biodistribution study in animals should be conducted, with dosimetry performed [49]. Consequently, the most recent interpretation of the EU regulatory framework for radiopharmaceutical testing and marketing approval considers non-clinical dosimetry data as essential information to be included in the investigational medicinal product dossier (IMPD) of a new compound [50, 51]. Some of the similar considerations apply to the United States legislation, where FDA expects that an IND contains “sufficient data from animal or human studies to allow a reasonable calculation of radiation-absorbed dose to the whole body and critical organs upon administration to a human subject” (title 21 CFR 312.23(a)(10)(ii) [3]). For diagnostics, it is also specified that preclinical models should be included; “the radiation safety assessment must establish the radiation dose of a diagnostic radiopharmaceutical by radiation dosimetry evaluations in humans and appropriate animal models” (title 21 CFR 315.6 (d), [3]). However, the 2014 EANM guidance document on how to prepare an IMPD for a radiopharmaceutical to be used in a clinical trial does not cover internal dosimetry [52]. A recent review published following an international meeting on “Preclinical testing of radiopharmaceuticals” supported by the International Atomic Energy Agency (IAEA) recognizes the relevance of preclinical dosimetry but, at the same time, acknowledges its lack of standardization [53]. In summary, in vivo preclinical pharmacokinetic studies of new radiolabelled compounds are almost invariably performed, whereas an estimation of human dosimetry based on biodistribution experiments on laboratory animals may not necessarily be available at the time a first-in-human trial is initiated.

In many cases, there will be similar radiopharmaceuticals already investigated in humans, such as when therapeutic compounds are developed following diagnostic tracers. Compassionate use cases of therapeutic compounds may also have been performed. This allows for initial estimates of source organs and measurement timing, after corrections for physical half-live differences are performed. It should also be noted that generic absorbed dose estimates of some classes of radiopharmaceuticals, for example, diagnostic brain receptor substances or amino acids, are given by the ICRP (e.g. ICRP 128) [44]. If highly similar tracers with the same radionuclide already exist, this information should be used as a starting point and evaluated during the first-in-human study.

It is important to mention that the well-established physiologically based pharmacokinetic-pharmacodynamic (PBPK/PD) modelling approaches for conventional drug development have only rarely been applied in radiopharmaceutical development. Both the EMA and FDA have guidelines on the reporting of PBPK modelling [54, 55]. A recent review focused on this aspect and came to the conclusion that the available literature would support the notion that these modelling concepts can result in a better understanding of PK and whole-body distribution of radiopharmaceuticals in general and could contribute to the evolving research of radiopharmaceuticals [56].

### Preclinical biodistribution and extrapolation

In the development of new radiopharmaceuticals, the translation from in vitro cell line uptake studies to small animal studies and finally into first in human investigations is a common path to define the pharmacokinetics (PK) and prediction of absorbed doses to humans [57]. This bench-to-bedside approach requires at several steps caution and harmonisations to prevent erroneous assumptions.

In preclinical biodistribution studies, a set of animals is administrated the radiopharmaceutical and the organ activities measured at several time points. This can be performed by sacrificing the animals and measuring tissues in well counters or by imaging the animal at multiple time points [58]. Investigation of PK and behaviour of new radiopharmaceuticals can also be done by using theranostic pairs to visualize the biodistribution by means of small animal imaging [59]. The imaging approaches require fewer animals. However, quantification may be challenging for smaller volumes and the procedures requires anaesthesia, which may alter the pharmacokinetics of the studied compound. This holds especially true for radiopharmaceuticals where the biodistribution depends on the level of specific hormones or neurotransmitters [60, 61]. In contrast, anaesthetics have virtually no impact on tumour targeting. With regard to predictive models of human pharmacokinetics, an initiative some ten years ago published some proposals how to proceed and summarized the potential pitfalls [62–66]. Besides anaesthesia, other important confounding factors in this context include fasting state, food-drug interactions, and circadian differences in metabolism [67]. All these aspects can result in significant impact on the extrapolation of the acquired results into the human situation, and as mentioned above, it is important to harmonize and to disentangle the potential impact.

Several methods have been described to extrapolate human absorbed doses from time-activity information obtained experimentally in small animals [68, 69]. The most straightforward approach often utilized is the direct application of the source organ time-integrated activity coefficient (TIACs) to human organ masses in order to calculate absorbed doses. However, owing to the differences in organ sizes and metabolic rates of physiological functions between species, power-law equations relating the variable of interest as a dependent function of the body mass, namely, allometric equations, have been developed in the pharmaceutical field [70, 71]. A popular approach, which is supported by some national guidelines such as the Swiss federal guideline [72], considers the application of a relative mass scaling factor to the animal TIAC to obtain the human TIAC [68, 73]. More complex allometric approaches have been proposed which consider time scaling and/or organ-specific metabolic scaling [7, 74]. These methods may provide different results depending on the characteristics of the radiopharmaceutical, and none has been universally adopted as the reference method for prediction of human absorbed doses, so far. Due to the various challenges, as briefly described above, it is important to realize that any animal model is at its best only an approximation of the human situation. It should be emphasized that any extrapolation should only be used as a starting point to inform the first-in-human study design, and cannot replace the study completely.

## Measurement protocol

**Fig. 3:**
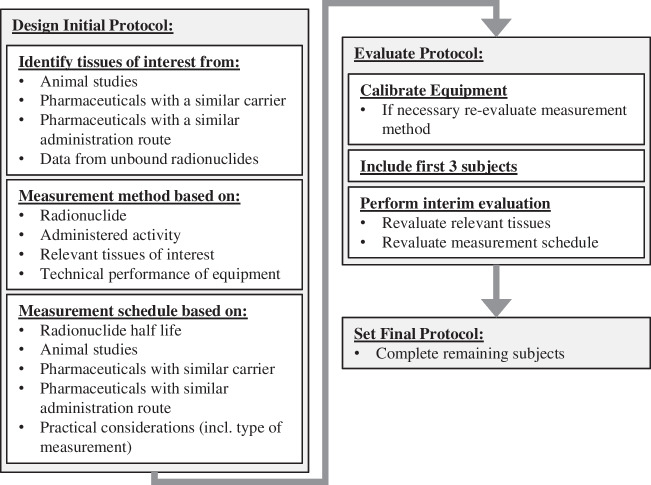
Flow chart for designing and updating the measurement protocol

The measurement protocol should be defined based on several factors (Fig. 3). A preliminary identification of source organs should be performed, as described in section “Identifying source volumes”. The kinetics of potential unbound radionuclides should be taken into account, and the relevant tissues where unbound nuclides are likely retained or tissues involved in excretion should also be included. The complete biokinetics of unbound nuclides for adults can be found in ICRP reports (the most recent versions are currently the ICRP 134, ICRP 137, ICRP 141, and ICRP 151) [75–78]. The uptake and clearance of potential radioactive daughters, metastable states, and other isotopes resulting from activated impurities during production should also be considered. A list of different measurement methods is given below, and choice among these will primarily depend on characteristics of the radionuclide, activity, and administration route.

Direct imaging of the radiopharmaceutical is preferred when technically achievable, also dependent on the uncertainty of the quantification procedure. When only select regions of the body are considered relevant for uptake analysis, and the primary focus of imaging, a whole-body overview should still be obtained to screen for unexpected behaviour—at least in an initial subset of participants. In addition to any imaging studies, whole body measurements with an external probe should always be performed as an efficient and reliable method to determine dose rates and whole-body retention over time [79]. If imaging proves challenging for the radiopharmaceutical (see section “Gamma camera imaging or SPECT/CT and PET”), diagnostic surrogate compounds may be considered [80].

Blood sampling is an essential part of first-in-human studies as it is needed for clearance estimates and can also be used for various dosimetric calculations such as for red bone marrow (see section “Organs with potential special considerations” [81]). If quantitative imaging is challenging, pharmacokinetic models in combination with blood samples can potentially be implemented. An alternative to assess activity in circulating blood, especially suited for diagnostic tracers with rapid uptake, is to perform imaging of the heart contents. This may also include a dynamic study for a few minutes during and after injection. The use of non-imaging detectors, e.g. above the heart or around the wrist have been suggested as a non-invasive alternative, but should be thoroughly validated before use in a first-in-human study [82]. Besides measurements of activity in blood, measurements of the carrier molecule and human in vivo stability may also be advised, especially for larger complexes such as antibodies or peptides [83, 84]. Biopsy studies may be considered for radiopharmaceuticals with uptake in bone marrow, although this will only inform the activity level at the sampling localisation [81, 85].

Temporal sampling should be selected based on both physical and biological aspects, as well as practical considerations. Report 67 of the International Commission on Radiation Units & Measurements (ICRU) [86] suggests measurements near 1/3, 2/3, 1 1/2, 3, and 5 times the effective half-life of the later clearance phase, and representing each biologic clearance phase and all major paths of excretion. If the effective half-life is not known, a conservative approximation is to use the physical half-life. While it is often desired to limit the amount of measurements in well-established clinical routine, it is recommended to allow for a more generous number in first-in-human studies since the biodistribution and kinetics will be more uncertain. Ideally, a minimum of three measurements per biological phase should be acquired in order to fully characterise the kinetics [87], and, if followed strictly, this would easily lead to at least six time points for most radiopharmaceuticals (and most often more as different source organs can have different kinetics). However, as the uptake phase can be fairly rapid, and hence contribute less to the time-integrated activity (TIA), it may be possible (or even unavoidable) to acquire less data for this phase. While multiple measurement techniques are often performed at the same time points, it should be considered whether this is strictly necessary and what is most practical. Unless it is desirable to directly validate one method against another (such as fluid samples extracted vs imaged-based content of hollow organs) [88], the formation of time-activity curve (TACs) for dosimetry purposes negates the need for truly simultaneous measurements.

It is recommended to design the protocol with flexibility to allow adjustments after an initial group of subjects (e.g. the first three) are included and preliminary analyses of this group performed. An example on this technique is provided in the example section (see Supplementary information). This facilitates both the reduction and optimisation of measurements within a protocol as knowledge of the biodistribution, and kinetics are established. A degree of flexibility is especially important to allow adaption in the imaging points without the need for a study amendment and further ethics approval.

For multicentre studies, standardisation of protocols and measurement output is highly important. Standardisation of output suggests that when needed, priority should be given to performance over establishing identical measurement protocols (same acquisition parameters, reconstruction algorithm, etc.). For example, for PET scanners, EARL, or other accredited protocols may be a sensible approach [32].

Similarly to the rest of the equipment used, the activity meter (often referred to as a dose calibrator or radionuclide calibrator) should be properly calibrated for the relevant radionuclide, activity level and geometry. Ideally, a traceable cross calibration to a primary standard of activity should be performed, which are often provided at a national level by National Metrology Institutes.

### Blood samples

Blood samples are typically drawn at multiple time points and measured in a gamma counter. The counter should be calibrated and characterised over the theoretical range of activity values. If the blood samples could contain contributions from multiple radionuclides of a decay chain, consideration should be given to using different energy channels or delayed measurements to separate the individual radionuclides based on half-life [89].

Further consideration should also be given as to whether whole-blood measurements are sufficient or if plasma or serum should be measured independently. Some compounds will bind to components in the blood (such as unbound lead to red blood cells [77]), and separating the compartments may then provide valuable information for establishing proper kinetics. Blood (or urine) samples can also be used to evaluate metabolic stability of the compounds by chromatographic techniques [83], preferably at several time points as the stability can change over time. This evaluation is recommended when the measured activity in fluid samples is used directly for dosimetric calculations, and the in vivo human stability unknown.

### Urine and faecal samples

The purpose of collecting and measuring activity within excrement is to indirectly estimate the retention within the body over time and/or for use as input for pharmacokinetic modelling or special dosimetric models. When knowledge of the total activity excreted is desired, this can be achieved by urine or faecal collection or spot samples in combination with diuresis measurements or weighing. Spot samples alone are more relevant for radiation protection considerations or verification of quantitative imaging approaches. The main route of excretion is also useful when ascertaining radiation protection requirements; an example of this is given in the [^223^Ra]RaCl_2_ example where faecal excretion was demonstrated to dominate over urinary excretion (see example section in supplementary information; “[^223^Ra]RaCl_2_”).

### Whole-body probe measurements

Choice of probe should be selected based on photon flux, for which a simple ionisation detector or a more sensitive scintillator-based probe may be appropriate. The suitability and properties of the probe for the intended radiopharmaceutical and the relevant time/activity span should be characterised before inclusion of subjects. Measurements at different fixed distances is advised, especially if the predicted biokinetics are uncertain or the expected input close to the operating range of the probe. In most cases, the first measurement of a subject post-administration serves as calibration for the whole-body activity at later time points, and this measurement should be performed before micturition.

### Gamma camera/SPECT and PET imaging

Depending on how well the performance characteristics of the imaging equipment for the given radionuclide are established, it may be necessary to first explore the potential for quantitative imaging by phantom studies [28]. For single photon emitters, the use of planar scintigraphy is generally suboptimal compared to SPECT imaging, in particular for overlapping organs [90], but can be useful for early phase dynamic studies [91]. Example approaches are suggested for the nuclides in Table 1. However, the radiopharmaceutical particulars may render the stated approach too uncertain for novel applications. Renewed calibration and imaging quality studies are then needed to determine the optimal imaging protocol. Investigations for dead time, noise equivalent count rate, and quantification uncertainties should be performed for all nuclides if the amount of activity to be imaged exceeds or is significantly less than the equipment’s previously established operating range for the specific radionuclide. This also applies if the structures to be investigated (i.e. source organs) is likely to be different in size or shape than previously charted structures, or the background activity or attenuating properties (e.g. anatomical size of the body part) may differ significantly. Typically, phantoms containing spheres of different sizes are used for such evaluations, but more anthropomorphic phantoms may also be considered [92]. Quantitative tomographic emission imaging enables the simultaneous collection of dosimetry-relevant information from multiple organs in a patient. However, different organs can present differing kinetic phases and/or different effective half-lives. Hence, the choice of the acquisition time points may represent a trade-off that should prioritize a more accurate TAC estimation of the most irradiated organs and/or organs at risk. It is recommendable to make sure patient positioning and imaging field of view are consistent between imaging time points.

### Anatomical imaging

Accurate estimation of individual tissue masses are in most cases essential for absorbed dose calculations for therapies. Anatomical imaging of diagnostic quality should then be available for all organs considered source volumes, acquired for at least one time point. Outlines drawn on CT or MRI in combination with tabular densities from, e.g. ICRP 89 or ICRU 46 [93, 94] may be used to derive organ masses. While stand-alone diagnostic scans can be used for this purpose, co-registered anatomical and nuclear medicine images provided by hybrid imaging systems (such as PET/CT or SPECT/CT) are beneficial to guide the delineation of activity. If delineated masks of organs based on anatomical imaging (or masks made from one of the nuclear medicine imaging time points) are used to derive the activity values, it must be considered that significant changes in patient positioning may take place between different image acquisition time points. This plays a particularly important role for small organs such as salivary glands, areas of high positional variability like the head and neck, or hollow organs such as bladder, stomach, and intestines.

## Biodistribution and time-integrated activity estimation

### Identifying source volumes

Source organs, i.e. organs characterized by significant transit of radioactivity due to the radiopharmaceutical uptake followed by physical decay and possible biological washout, should be initially identified based on pre-clinical data, and existing clinical information collected from in-human studies involving radiolabelled compounds with expected similar biodistribution (Fig. 3).

As a rule of thumb, major parenchymal organs such as the liver, lungs, kidneys, and spleen should always be considered as potential source organs in a first-in-human study. Similarly, the blood pool should be included, as well as organs involved in the excretion pathways such as the various parts of the gastro-intestinal tract and the urinary system including the respective contents. Due to the comparatively high radiosensitivity of red bone marrow, it is generally recommended to consider this as a potential source organ, even if specific uptake is not initially identified. For loco-regionally delivered radiopharmaceuticals (see section “Administration route”), systemic leakage should not be disregarded. For example, using microspheres for selective internal radiotherapy of disease in the liver, lung shunt has been found a potential limiting factor [20].

Depending on the specific biodistribution, other tissues should also be considered source organs. A non-complete list includes adrenal glands, brain, cortical bone, eyes, gallbladder contents, heart wall, ovaries, pancreas, prostate, red bone marrow, salivary glands, testes, thymus, thyroid, trabecular bone, and uterus. These tissues are commonly available as source organs for input of TIA in most organ-based dosimetry software, for example, Olinda EXM, IDAC, OpenDose, and MIRDcalc [95–99]. Many of the tissues are also among the listed target organs in the output. (Note that “target organs” here refers to organs for which absorbed doses are calculated in the context of safety, not to be confused with, e.g. targets for a treatment.) If tissues of interest are not included in standard organ-based dosimetry software, dedicated absorbed dose calculations should be performed. An example approach, used for the choroid plexuses for [^68^ Ga]Ga-NODAGA-RGDyK [100, 101], is described in the example section (supplementary information).

### Curve fitting

The activity (or the activity concentration) present in source organs at different time points is required for the dosimetry workflow, as the absorbed dose calculations consider the energy deposited in target volumes based on the radiations emitted from source organs. As described in “Measurement protocol”, a sufficient number of measurements have to be available to appropriately characterize the kinetics in source organs and derive TACs. TAC fitting is a crucial step for computing accurate source-organ TIACs. Numerical and/or analytical time integration of source organ TACs provides the TIA. TIACs are then obtained by dividing the TIA by the total administered patient activity. In general, three models may be used for mathematically describing the biodistribution of a radiopharmaceutical over time, analytical models using a sum of exponential functions, empirical models, or compartmental models [102]. Mono-exponential analytical fitting is suited for tissues presenting TACs which can be described by a single clearance phase with a prompt initial uptake and also for whole-body (WB) retention fitting. In general, biological processes are expected to follow first-order kinetics, and multi-exponential TAC fitting can then describe the biodistribution and pharmacokinetics in source tissues characterized by multiple phases (e.g. fast uptake, fast and slow clearance). Model selection can for example be performed with the corrected Akaike information criterion (AICc) [103]. Linear interpolation followed by trapezoidal integration is a common empirical model which should be avoided if possible. However, it can offer a valuable solution when the TAC (or parts of the TAC) does not present any exponential behaviour. A disadvantage is the need to extrapolate information for the period after the last measurement (also before the first measurement if the initial conditions are undefined). Two methods are generally adopted to evaluate the late TIA contribution. The first method is a most often conservative approach that extends the TAC to infinity by considering only physical decay. The second method considers the TAC tail beyond the latest acquired time point as prolongation of the late clearance phase (represented by the effective half-life from the last measurements) to infinity. In this case, one must verify that the late effective half-life does not exceed the physical radionuclide half-life. Only in absence of a realistic late effective half-life should the “conservative” physical decay approach be used. In either case, the fraction of the TAC that is extrapolated should be reported. Compartmental models use differential equations to describe the transfer of radiopharmaceuticals between compartments of a system, often at organ or sub-organ level. While in silico models in theory could replace first-in-human studies, data size can be a challenge for the development of models for most first-in-human studies. However, in some cases, a priori knowledge can be integrated. For transfer rates, this requires previous studies of similar carriers in humans. A highly relevant scenario is the presence of unbound radionuclides, in which biokinetics of the radionuclides can be derived from ICRP publications as described [75–78].

Regardless of the fitting approach, a larger number of acquisitions generally improve the accuracy in estimating the actual tissue TAC and hence the absorbed dose. Different publications have demonstrated the importance of well-selected time points, especially considering late data acquisitions, in providing accurate TIA estimates [17, 87, 104, 105]. By the nature of first-in-human studies, the initial temporal sampling may not represent the optimal schedule. If one includes an initial cohort of subjects (Fig. 3), the evaluations performed for investigated fit models and selection criteria for this population should be reported. However, other measurement time points, resulting in other best fit models, may be used for the remaining subjects. The final TIA values should therefore ideally be established first after evaluations of the entire cohort are performed, to ensure consistency.

## Absorbed dose calculation

Dose calculations should be undertaken following the system of radiological protection described by the ICRP (ICRP 53, ICRP 103) and the dosimetry schema presented in the MIRD publications [106–109]. Given the different endpoints and causalities of risk between diagnostic and therapeutic studies, it is recommended that two differing approaches to absorbed dose calculation and reporting are used.

### Definitions

#### Absorbed dose, ${\varvec{D}}$

The absorbed dose is the energy absorbed per unit mass, and its unit is joule per kilogram (J/kg), which is given the name gray (Gy). Absorbed dose is defined in terms that allow it to be specified at a point, but can also be given as the average dose over a target tissue or organ $T$, written ${D}_{T}$, where $d\overline{\varepsilon }$ is the mean energy imparted to matter of mass $dm$ by ionising radiation:

$$D=\frac{d\overline{\varepsilon }}{dm}$$

#### Equivalent dose, ${{\text{H}}}_{{\text{T}}}$

This is the absorbed dose averaged over a tissue or organ and weighted for the radiation quality of interest. The equivalent dose in a tissue or organ T is given by the following:

$${H}_{T}=\sum_{R}{w}_{R}{D}_{T,R}$$

where ${D}_{T,R}$ is the mean absorbed dose from radiation R in a tissue or organ T and ${w}_{R}$ is the radiation weighting factor. The value of the radiation weighting factor for a specified type and energy of radiation is selected to represent the relative biological effectiveness of that radiation in inducing stochastic effects (e.g. cancer) at low absorbed doses. For gamma and beta emissions, ${w}_{R}$ is given the value 1. For α-particles, the ICRP consensus recommendation for ${w}_{R}$ is 20 [110]. This formalism is only applicable for stochastic effects, and its application in therapeutic settings is non-sensical. The use of equivalent doses for patient exposure should thus be limited to diagnostic settings. The unit for equivalent dose is the same as for absorbed dose, J/kg, but given the name sievert (Sv).

#### Effective dose, ${\text{E}}$

Effective dose is a quantity aimed to combine the different doses that organs of the body receive and correlate them with the total stochastics effects of such irradiation. Effective dose is therefore defined as the sex-averaged, tissue-weighted sum of the equivalent doses in all specified tissues and organs of the body considered sensitive to the induction of stochastic effects, given by the following expression:

$$E=\sum_{T}{w}_{T}\left[\frac{{H}_{T}^{M}+{H}_{T}^{F}}{2}\right]$$

where ${w}_{T}$ is the tissue weighting factor, which represents the relative contribution of the organ T to the total probability of stochastic effects. ${H}_{T}^{M}$ and ${H}_{T}^{F}$ are the male and female equivalent organ doses, ideally calculated from male biokinetic data in conjunction with the male reference phantom and female biokinetic data with the female reference phantom respectively. If insufficient data are available to create sex independent biokinetic models, then a single model can be used in both reference phantoms. However, it is important to state when this approach has been taken. The unit for the effective dose is the same as for absorbed and equivalent dose, J/kg, and is named sievert (Sv), similar to ${H}_{T}$. Revised values for ${w}_{T}$ are given in ICRP 103 [106] and summarised here in Table 3. The tissue weighting factor of 0.12 for the remainder tissues applies to the arithmetic mean dose of the 13 organs and tissues listed. Similar as for equivalent dose, evaluation of effective dose should be limited to a diagnostic setting.

**Table 3:** Tissue weighting factors according to ICRP 103 [106]

Tissue	${w}_{T}$
Red marrow	0.12
Colon	0.12
Lungs	0.12
Stomach	0.12
Breast	0.12
Gonads	0.08
Bladder	0.04
Oesophagus	0.04
Liver	0.04
Thyroid	0.04
Bone Surface	0.01
Brain	0.01
Salivary glands	0.01
Skin	0.01
**Remainder tissues** (adrenals, extrathoracic region, gall bladder, heart, kidneys, lymphatic nodes, muscle, oral mucosa, pancreas, prostate, small intestine, spleen, thymus, uterus/cervix)	0.12
**Total**	**1.00**

### Therapy

For therapeutic radiopharmaceuticals, the mean absorbed dose to the organs or sub-organs that demonstrate activity accumulation should be calculated and reported for individual patients. The absorbed dose is the primary parameter responsible for the biological effects observed, both efficacy and toxicity. Available evidence indicates sigmoidal correlations for the absorbed doses required to induce biological effects in both tumours and healthy tissues. Beyond certain thresholds, an increase in activity may therefore be accompanied by additional toxicities without providing any major therapeutic benefits.

Absorbed dose calculations should be performed using patient-specific kinetic data and patient-specific anatomy. Mass scaled, model-based S-values may be used when direct radiation transport simulations using patient-specific anatomy are unavailable. If organ mass is determined empirically, the methodology should be clearly stated and justified. Lack of published or reference S-values for an organ or target should not negate the requirement to calculate the absorbed dose, and an appropriate modelling method should be used as an alternative. Unless modelling the cross dose to the target organ, reference masses of non-source organs should be used for model-based approaches. Mean absorbed doses to the total body and to typical organs at risk, such as bone marrow or kidney, should also be reported irrespective of active uptake. Alongside mean organ absorbed doses, other dosimetric parameters such as percentage of injected activity (%IA), mean TIAC, and parameters describing the kinetics, such as mean effective half-life, should be reported. In addition to mean organ absorbed doses, dose calculations at the voxel level might also be given, provided that robust methods for image registration and resolution modelling are used to minimise fitting- or resolution-related errors [111]. Examples of, e.g. the time-activity curves at the voxel level should also be reported to demonstrate the validity of the fits. The penetration range of the emissions should be considered with respect to whether Monte Carlo–based voxel-level dosimetry is needed or if kernel or multi-kernel methods can be used.

### Diagnostic tracers

Evaluation of the stochastic risk from a new radiological test is often made in comparison to that of similar exams or approaches. For diagnostic applications, the ICRP system of protection is based on the use of standard reference anatomical and physiological models. The absorbed doses to individuals therefore have less relevance and the absorbed doses calculated using absorbed fractions derived from the standard reference phantoms should either be used or reported in conjunction with patient-specific doses. The effective dose is designed by ICRP to be calculated using the absorbed fractions derived from the standard phantoms (ICRP 133) [112], and should not be calculated using individual anatomical data. These S-values are also available in software such as Olinda/EXM 2.0, IDAC and MIRDCalc.

The effective dose is used to estimate the radiation detriment to a population, averaged over the full age distribution and for an equal number of both sexes. Therefore, in a biodistribution study, a sufficiently large sample of both male and female patients across the weight class of the ICRP reference phantoms should be included. The age distribution should also be considered.

When determining the effective dose, the TIACs assigned to the male and female reference phantom should ideally be based on a pharmacokinetic model using decay data averaged from the study cohort. TIAC values can also be evaluated using the mean organ concentration of the population and total organ activity scaled to the mass of the phantom model. If taking this approach, care should be taken to ensure that 100% of the activity is accounted for, which may require using a “remainder of body” compartment such that the summed activity in all source organs equates to that measured in the total body. Effective dose is conventionally reported using the sex-averaged equivalent doses calculated using the respective phantoms.

### Organs with potential special considerations

Some tissues may require special considerations or models. A non-complete list include various hollow organs, bone marrow, lesions and other small structures, or sub-compartments of organs, for which some examples are given in the following.

In general, while the absorbed dose to the contents of a hollow organ is not of interest, potential filling and excretion schedules may influence the doses to surrounding tissues. Examples of hollow organs include the urinary bladder [113], the gastro-intestinal tract [114–117], and the peritoneal cavity [118]. For beta- or photon contributions, models incorporated within OLINDA/EXM 2.0 may be used for absorbed dose estimations. Assumptions related to the models, e.g. voiding schedule, should be stated in the dosimetry report.

Special considerations for red bone marrow and dosimetric approaches have been previously described in an EANM guideline [81]. In brief, blood-based approaches can be utilised as long as there is no specific uptake in the region; if so, quantitative imaging needs to be performed. The underestimation that can occur using an incomplete model has been demonstrated for several radiopharmaceuticals, including one of the provided examples: [^177^Lu]Lu-lilotomab satetraxetan (see Supplementary information) [119]. The dose estimates will also depend on the fraction of red marrow [120], and the approach to estimating cellularity should be reported.

For sphere-like structures, e.g. lesions or small organs such as lacrimal glands, the absorbed self-dose can be estimated by spherical models of various emitters and sphere sizes [121].

In general, differences in TIAs or assumed radiosensitivities for sub-compartments of tissues, such as the renal cortex and medulla [122, 123], may necessitate separate small- or micro-scale investigations to provide clinical relevant information. Since such approaches are likely to be based on extrapolations, the assumptions should be clearly stated in the report. Small- or micro-scale investigations will also need to be evaluated in relation to the range of the radiations and is hence contextualised by the recent increase in alpha-emitter based treatments (Fig. 1).

### Uncertainty estimation

When reporting absorbed dose values, it is important to know the uncertainty associated with the calculated result. This uncertainty aims to characterize the range of values within which the true value lies, specified with a stated level of confidence. The EANM practical guidance document on uncertainty analysis [4] recommends an uncertainty schema that addresses many of the systematic and random sources of error in a dose calculation. Important aspects include the uncertainty of each TAC value and the appropriateness of the fit function [124, 125] and other fundamental aspects such as the uncertainty associated with system calibration, image registration, and volume-of-interest (VOI) delineation. Reporting of uncertainties in first-in-human dosimetry studies is currently often lacking. However, given the potential impact these data have, both from a legislative, clinical, and radiation protection perspective, it is arguably most important in this regard to have an indication of the reliability of the results.

A source of uncertainty that can easily be reported and potentially reduced is that associated with the timing choice of activity measurements. The goodness-of-fit is a straightforward method allowing an uncertainty estimate of the fitting parameters and TIA to be reported. This is potentially one of the most important aspects for first-in-human dosimetry, where the pharmacokinetics of the new radiopharmaceutical and hence optimal timing of the dosimetry measurements are largely undefined. Thus, an interim analysis of uncertainty and the influence of individual measurement time points can be used to optimise and potentially reduce the measurements regimen for dosimetry in subsequent patients or cycles. In the diagnostic setting when determining biokinetics of a study cohort, uncertainty can either be determined by performing the fitting analysis on all participant data points simultaneously or by using the average of the cohort for each time point (assuming consistent timings across the population). In the former approach, the uncertainty in fitting can be determined using an ordinary least squares fit (assuming equal weighting of all data points). In the latter approach, an uncertainty for each time point can be determined from the population distribution and a generalised least squares approach used to propagate this into uncertainty in the fitting parameters [126].

The accuracy and traceability of activity concentration in the body are significant factors for dosimetry applications. For SPECT/CT-based dosimetry, the accuracy of the activity measurement is largely dependent on the accuracy of the radionuclide calibrator used to cross-calibrate the imaging system. Therefore, this should ideally be traceable to a national metrology institute and quoted as part of the uncertainty report. In the context of multi-centre, first-in-human dosimetry, harmonization of activity measurements is a key aspect to ensure that the dosage information obtained at an individual site can later be used for implementation and/or improvement of the therapy at other sites.

### Reporting

A summary of parameters to be reported is provided in Table 4. Methodological descriptions and qualitative considerations that should accompany the report are described in the following. Summary statistics including mean, standard deviation, and range of absorbed doses should be provided, even though the population sizes are normally too small (< 100) for what is commonly assumed reliable standard deviations. The values should be provided for each investigated tissue, in addition to WB. The method used to select source volumes should be described, and if multiple methods have been investigated, the results should be compared taking the uncertainties of each approach into account. For FDA or EMA approval, safety estimations for a “complete” set of human organs are traditionally required. For example, the list of target organs in the ICRP 128 [44] includes adrenals, bone surfaces, breast, brain, gallbladder wall, gastrointestinal tract, heart wall, kidneys, liver, lungs, oesophagus (thymus), ovaries, pancreas, red marrow, skin, spleen, testes, thymus, thyroid, urinary bladder wall, uterus, and other tissues, with the possible addition of lachrymal glands, salivary glands, and spinal cord. For diagnostic tracers, equivalent dose and effective dose should also be reported using the most recent radiation and tissue weighting factors. The sets of weighting factors used should be stated. Considerations should be given to including reports of effective dose using older factors for comparison purposes. For treatments with alpha-emitters, relative biological effectiveness (RBE) weighted absorbed doses can be calculated [110], although this is currently not recommended as the primary parameter to be reported due to uncertainty of the RBE-values. The choice of RBE value should be stated indicating its biological end-point and ideally also justified if this is included in addition to the absorbed doses. The separate contributions from all nuclides and radiations to the total absorbed dose should be listed if applicable. Reporting of biological effective dose (BED) is encouraged for therapies. However, the choice of radiobiological formula(s) and parameters should be stated. Ideally, a range of relevant radiobiological parameters should be explored if the exact values are unknown. Similar considerations for normal tissues apply to tumours or other regions of disease, which should also be included in the report if feasible. Absorbed doses for sub-structures or voxel-dose-maps can be reported if providing relevant clinical information. Volume cut-offs such as D90% or D98% can also be reported as summary statistics for relevant tissues. In accordance with principles of findability, accessibility, interoperability, and reusability (FAIR), all dose parameters to individual participants, as well as all activity measurements for each time point, TAC parameters (such as effective half-life), and potentially the volumes should be made available. Uncertainty estimates should be included for the sources investigated, and potential sources not investigated described. In addition, information about all underlying assumptions, details of acquisition or measurement settings, factors related to the radiopharmaceutical, as well as details of the dose calculations themselves [5] should be reported. How and why the final dosimetry study protocol was adapted based on the initial subject cohort should be detailed, if relevant. In cases where dosimetry planning is performed prior to a therapy, both the predictive and actual dosimetry results should be reported. This could insights of the validity of the planning approach, but also about the assumptions made increasing the information gained within the trial. When available, the results from the first-in-human dosimetry study should preferably also be compared to any available preclinical data of similar radiopharmaceuticals, contributing to a foundation for improving translational knowledge regarding biodistribution and dosimetry for radiopharmaceuticals.

**Table 4:** Summary of recommendations for parameters to be reported

Parameter(s)	Recommendation, diagnostic tracers	Recommendation, therapies
Absorbed dose for all source tissues and WB	Should be reported as population summary statistics including mean, standard deviation and range AND Should be reported for individual subjects including activity measurements at each time point, TAC parameters, and potentially volumes	Should be reported as population summary statistics including mean, standard deviation and range AND Should be reported for individual subjects including activity measurements at each time point, TAC parameters, and volumes
Absorbed doses to target tissues representing a complete set of human organs	Should be reported as summary statistics and for individual subjects	Should be reported as summary statistics and for individual subjects
Equivalent dose and effective dose	Should be reported using population data and most recent radiation and tissue weighting factors If deemed relevant can also be reported using older sets of weighting factors	Non-sensical
Dose parameters for tumours or other regions of disease	Not relevant	Should be reported for individual regions, and as summary statistics for tumour type. Should include activity measurements at each time point, TAC parameters, and volumes
Dose parameters for organ sub-structures	Not relevant	If deemed relevant, to include activity measurements at each time point, TAC parameters, and potentially also volumes
Relative biological effectiveness (RBE) weighted absorbed doses for source tissues and/or regions of disease	Non-sensical	Optional (only for alpha- or auger-emitters)
Biological effective doses (BEDs) for source tissues and/or regions of disease	Non-sensical	Encouraged, reported for each individual and as summary statistics
Voxel-dose-maps, including volume cut-offs (such as D90% or D98%) for summary statistics	Not relevant	If deemed relevant

## Radiation protection considerations

In many situations, differences concerning radiation protection between a “new” radiopharmaceutical and radiopharmaceuticals already in use will be minor. When introducing new tracers or treatments, previous risk assessments, information sheets, and written instructions should constitute a useful framework for development prior to gathering further knowledge from the biodistribution study.

New radiotracers for imaging may be tested on volunteers, for whom national or local ethical committees will have specific dose constraints. By nature of the studies (“first in human”), the compliance with these can in advance only be based on extrapolated information from similar tracers or from animal studies. The relevant amount of activity can most often be estimated from data of similar tracers and potentially adjusted after the first pilot studies. Even if an unexpected high activity is found in a single organ, this is not likely to create any harm, but may only limit the recommended activity applied for future use. For therapies, the situation is different (always performed in patients), and some scheme of activity dose escalation will normally have to be applied, enabling dose calculations to be performed at varying levels of injected activity.

Radiation properties of new radionuclides must be considered to ensure that existing shielding, handling and ventilation equipment, etc., is sufficient for protection of staff. This should include all emissions, even those not directly relevant for the intended application. Some nuclides, for example, zirconium-89, are intended for PET, but also emit photons with gamma energies that are significantly more penetrating than the standard annihilation energy. Nuclides with high beta particle energies can give excessive finger (or eye lens) doses, and handling tools must be optimized to limit that risk. For example, shielding may have to be optimized with subsequent layers of low and high atomic numbers to minimize the production of bremsstrahlung radiation. For alpha emitting nuclides, the focus should in particular be on limiting any risk of the activity becoming airborne and inhaled, because the dose factors for inhalation or intake can be very high.

Patients or volunteers receiving activity for diagnostic purposes can usually be released shortly after the examination. However, in the context of the dosimetry study, it may be preferable that they stay for longer durations. For therapy patients, the time to remain in the hospital depends on rules and regulations that may vary between different countries and regions. Radiation protection considerations should then also be given to the patients’ travel to and from the institution during the study.

A risk assessment must set dose constraints for patients’ carers and comforters in accordance with the basic safety standards directive [127]. Also for members of the public, it should be documented that the released patient is not likely to infer doses over a value set by local authorities, typically a fraction of the yearly dose limit of 1 mSv. The potential irradiation of patients’ family members or colleagues must also be assessed and described in the information provided to the patient together with specific recommendations for distance and time. Specific recommendations for contact with children or pregnant women should also be provided if relevant. The consequences for burial or cremation after eventual death of the patient should be considered. For the predominantly short-lived nuclides applied in diagnostic imaging, exposure of family or members of the public is not likely to be an issue. For radionuclide therapy, this is important information that must be updated as the results of the study evolve.

The amount of activity and the route of excretion must be determined in the study, not only for dosimetric purposes, but also to ensure that local rules of discharge are followed. Some countries’ legislation or institutional practices requires the use of collection tank systems for delay and decay, while others may rely on dilution and dispersion in the sewer system. For new radionuclides and radiopharmaceuticals, calculations for tank capacity as well as shielding and direct discharge amounts should be updated as excretion data is gathered and the radiological risk assessment updated accordingly, also taking into account potential long-lived metastable states or impurities.

## Clinical implications and conclusions

It is important to consider that dosimetry for first-in-human studies is not only a legislative requirement, but can also provide relevant information for the continued development of the diagnostic investigation or therapy. For this purpose, it is strongly encouraged that results from all first-in-human dosimetry studies be made publicly available and published in a peer review journal, making use of supplementary material to provide a full and complete presentation of the data described above.

For diagnostic tracers, comparisons of effective doses to other imaging options can aid in the selection of the most appropriate investigation for a given clinical situation. The quantitative measurements used to determine the biodistribution can also guide in selection of the optimal imaging time point and/or injected activity. For treatments, the range in absorbed doses for individual normal tissues should be considered together with existing evidence of dose–effect correlations and radiobiological parameters for the tissues to establish the limiting organ(s)-at-risk. Much of this data still remains to be confirmed on a larger number of patients and radiopharmaceutical products. Clinical radiobiology approaches based on the analysis of patient samples (tumour biopsies and blood samples) can also contribute to this knowledge, along with factors beyond the dose [128, 129]. Theoretically, the prescription basis for a therapy could be determined without dosage escalation studies as long as the prerequisites are firmly established. While it is not explicitly required by the legislation for early phase studies, absorbed dose to tumours should preferably also be determined so long as it is technically achievable. Dosimetry could then be used to identify the “therapeutic window” between destroying cancer tissue and preserving healthy tissues. If different treatment schemes are investigated, the results may be used to indicate at an early stage—before clinical follow-up data are available—which one is preferred. Furthermore, many studies have been performed using a traditional activity escalation design, which tends to lead to undertreatment by precaution [130]. Dosimetry-based regimens can then contribute to increased opportunities for treatment individualisation. Considerations of how this can best be performed and implemented in clinical routine or later trials are warranted for each individual study.

## Liability statement

This guidance document summarizes the views of the EANM Dosimetry Committee, written in collaboration with authors from the EANM Radiation Protection, Translational Molecular Imaging and Therapy, Radiopharmaceutical Sciences, and Oncology and Theranostics Committees. It reflects recommendations for which the EANM cannot be held responsible. The recommendations should be taken into context of good practice of nuclear medicine and do not substitute for national and international legal or regulatory provisions.

### Supplementary Information

Below is the link to the electronic supplementary material.

Supplementary file1 (PDF 1006 KB)

## Abbreviations

AICc: Corrected Akaike information criterion
BED: Biologically effective dose
EMA: European Medicines Agency
FDA: Food and Drug Administration
IAEA: International Atomic Energy Agency
ICRP: International Commission on Radiological Protection
ICRU: International Commission on Radiation Units & Measurements
IMPD: Investigational medicinal product dossier
IND: Investigational new drug applications
MIRD: Medical Internal Radiation Dosimetry
PBPK: Physiologically based pharmacokinetic
PK: Pharmacokinetic
RBE: Relative biological effectiveness
TAC: Time-activity curve
TIA: Time-integrated activity
TIAC: Time-integrated activity coefficient
VOI: Volume of interest
WB: Whole body
