# Supplementary material for: EANM guidance document: dosimetry for first-in-human studies and early phase clinical trials
Source: Eur J Nucl Med Mol Imaging. 2024 Feb 17;51(5):1268–86. doi: 10.1007/s00259-024-06640-x (PMC10957710; doi:10.1007/s00259-024-06640-x)
Supplement: Supplementary file 1 — Supplementary file1 (PDF 1006 KB) [file 259_2024_6640_MOESM1_ESM.pdf]

Examples - Supplementary Material to EANM guidance document: Dosimetry for first-in-human studies and early phase clinical trials

The following section gives three examples of first-in-human dosimetry studies. These examples are not given to demonstrate best practice but rather illustrate the different approaches that may need to be adopted for different radionuclides. Each study highlights potential difficulties faced by the investigators and how these were tackled.

[^{177}Lu]Lu-lilotomab satetraxetan for non-Hodgkin lymphoma (NHL)

The first phase 1/2a study of [^{177}Lu]Lu-lilotomab satetraxetan, for relapsed CD37+ indolent NHL, started in 2012 (NCT01796171) [1]. The treatment was antibody-based and targeted the internalizing CD37 antigen, which is expressed on B-cells. Different patient arms with various pre-dosing and pre-treatment with both anti-CD37 and anti-CD20 were investigated. The initial imaging and measurement protocol contained planar WB scans acquired approximately 2, 4, 8, 24, 96, and 168 h p.i., and SPECT/CT 96 and 168 h p.i. [2]. Blood samples were collected several times on the same day as [^{177}Lu]Lu-lilotomab satetraxetan was administrated (day 0), once on days 1, 2, 3, 4, and 7, and every week thereafter until week 12. After the first four patients (arm 1) had been included, the imaging and dosimetry protocol was evaluated and adjusted. Below, some of the considerations and adjustments performed at various stages are summarised.

<u>Set-up of the initial measurement protocol:</u>

- Antibody-based treatment and the physical half-life of lutetium-177 suggest measurement time points spanning at least a week after injection.
- Dosage starting at 10 MBq/kg body weight, which was translated from preclinical investigations. This relatively low amount of activity suggested prolonged image acquisition (45 min per SPECT bed position). Only the 208 keV peak was used for quantification, and an additional reconstruction was made for visual analysis, combining the 113 and 208 keV photon peaks.

<u>Evaluations of initial protocol after the first four patients were included:</u>

- Number of imaging time points was reduced, since it was determined that only one of the initial three time points at the day of injection was needed to estimate the time-integrated activity [2], see also fig x1. The choice of time point to keep (2 hours p.i.) versus the time points to omit (4 and 8 hours p.i.) was primarily due to logistics.
- Transition to dosimetry based on SPECT only. First, planar images were investigated after attenuation correction and background correction [2]. However, as several source organs had overlap or low amounts of activity contained, this method could lead to "non-physical" values (effective half-life > physical half-life for the wash-out phase).

<u>Additional evaluations and validations:</u>

- Imaging at additional time points was performed for a few patients in order to validate the TAC models (e.g. 14 days p.i.).
- It was suggested to investigate red bone marrow dosimetry by the blood surrogate method, which proved to underestimate the red marrow absorbed dose [3]. This was expected as specific uptake was observed in the marrow. The surrogate method also failed to show any correlations with haematological toxicity (as the SPECT-based method demonstrated).

- Different approaches were investigated, both to extract additional information (e.g. voxel-based dosimetry for tumours [4]), and to simplify the quantification procedure (use of “small-VOIs” instead of complete organ delineation).

Clinical implications:

- The ratio between tumour and red marrow absorbed dose increased for the patient arms including pre-dosing with cold lilotomab antibodies [5], which contributed to the continuation of these arms in phase 2a.
- Red bone marrow was found to be the limiting normal tissue for this treatment, and the absorbed doses to marrow correlated with haematological toxicity [6]. While the range in absorbed doses both for red marrow and tumours [7] suggested an individual treatment planning approach, repeated injections were to be avoided at the time. Later preclinical developments have included a humanised antibody and a diagnostic companion (anti-CD37 carrying zirconium-89), which could have allowed for such approaches [8, 9].

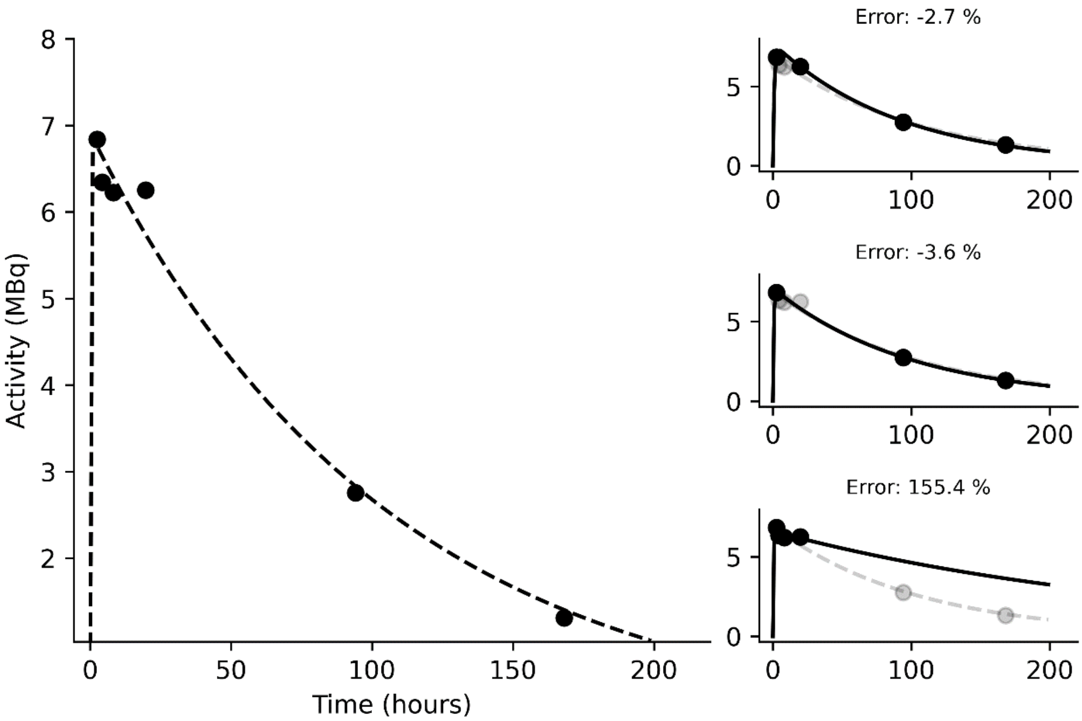

Figure x1. Examples of time-activity curves (TACs) for kidneys obtained using the initial (left panel) and simulated imaging protocol for [^{177}Lu]Lu-lilotomab satetraxetan. The simulated panels show the deviation produced by removing various time points from the initial protocol before the time-integrated activity coefficient (TIAC) calculation. The average of four patients is shown. The upper right simulated panel shows the protocol used for subsequent patients.

[^{223}Ra]RaCl$_2$

[^{223}Ra]RaCl$_2$ is a bone-seeking alpha emitter for patients with bone metastases from castration resistant prostate cancer. The phase 1 Open-label, Dosimetry, Biodistribution and Pharmacokinetic Study of Alpharadin (later marketed as Xofigo) took place between July 2007 and December 2008 (NCT00667537). Six participants were recruited into this study with the primary outcome measure to estimate whole-body, organ and blood retention, urine and faecal elimination, and pharmacokinetics and absorbed doses to organs. A secondary aim was to compare the biodistribution and dosimetry between the two administration cycles. ^{99m}Tc-methylene diphosphonate bone scans were acquired at pre-treatment assessment. The therapy protocol followed with two intravenous injections of [^{223}Ra]RaCl$_2$ administered 6 weeks apart at an activity of 100 kBq per kg of body weight. Patients remained in the hospital for approximately 48 h after each administration.

Previous Data

The ICRP had previously developed a biokinetic model for radium and other alkaline earth elements based on the model for adults proposed by Leggett [10, 11]. The biodistribution of radium-223 had also been studied in mice prior to a therapeutic efficacy study in a nude rat model [12, 13], and the first clinical experience had been documented by Nilsson et al in 2005 [14]. In this first-use study, blood clearance was measured up to one week post administration and, despite the low intensity of gamma emissions from radium-223 and its daughter nuclides, the first planar scintigraphic images were acquired.

Study Workup

Although the proportion of gamma emissions from each radium-223 decay is low, scanning and counting of patients and samples was shown to be feasible [14]. However, a well-defined quantitative imaging protocol was not available. A pre-patient phantom study was therefore undertaken to determine the gamma camera imaging parameters required to optimize radium-223 imaging. Clinically realistic measurements were used to characterize sensitivity and spatial resolution and ascertain the optimal collimator and energy window settings. The accuracy of quantitative imaging was then verified in phantom studies [15].

Measurement Protocol

The initial imaging and measurement protocol was developed based on the pre-existing pharmacokinetics models. Blood extraction was performed immediately after injection; then at 15, 30, and 45 min and 1, 2, 4, 24, 48, 96, and 144 h after injection. Activity in the whole blood and plasma were independently measured. Pre-injection and first void urine sample were collected separately, followed by total urine output for the periods 0–4, 4–8, 8–24, and 24–48 h after injection. All faeces excreted by patients were collected up to 48 h and measured using gamma spectroscopy. Similarly, gamma spectroscopy measurements of the whole body were taken immediately after injection, before first void, at 1 hour, and thereafter every 2 hours during the first day, then at least twice daily until discharge. Subsequent measurements were taken at 96 and 144 h.

Due to the low yield of gamma emission and limitations in the imaging technology at the time, SPECT/CT was deemed unfeasible. WB images were therefore used for activity distribution imaging and to determine quantitative uptake in organs. Imaging was performed using a medium energy general purpose collimator and an energy window set at 82 keV with a 20 % width to encompass counts from the 81- and the 84-keV emissions. The first scan was acquired within 0–4 h after injection, and subsequent scans acquired at 24, 48, 96, and 144 h after injection. Example retention and excretion data for a single patient are shown (fig. y1).

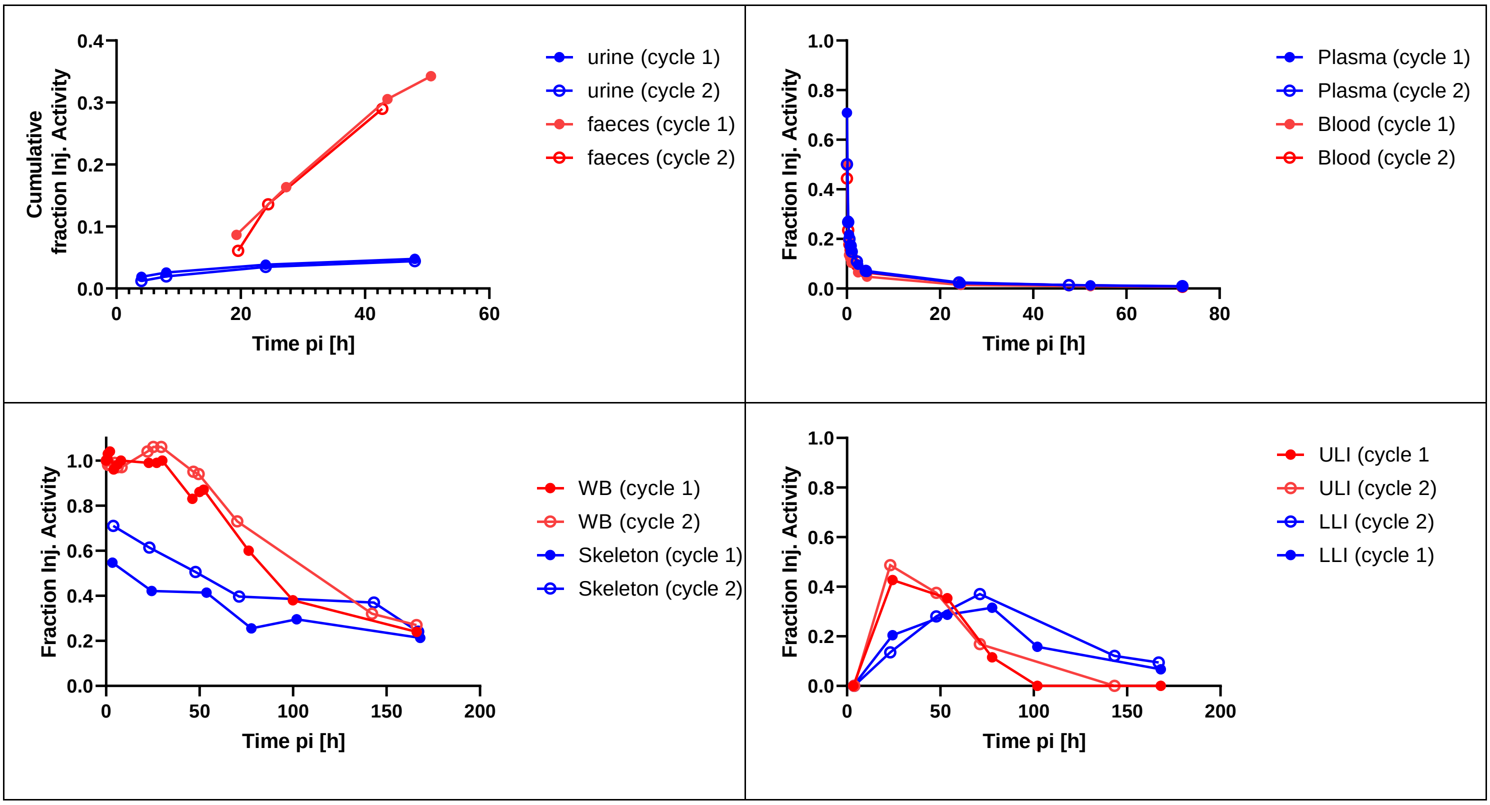

Figure y1 Example time-activity curves (TACs) for different organs and compartments of the body. Data shown for a single participant for each therapy cycle.

Dosimetry

For imaged organs, TIAs were calculated by trapezoidal integration assuming activity at time t = 0 was that at the first measurement point. The effective half-life as determined from the last measurement time point was used for extrapolation to infinity.

Olinda S-values were used, taking into account the emission and branching ratios of all daughter nuclides. As kidneys were not visible on the scintigraphic images, kidney absorbed dose was calculated from urine concentration measured and assumed to be uniformly distributed within the kidney. Marrow dose was calculated assuming an equal concentration of activity in the marrow to that measured in the blood.

The absorbed doses to normal organs were calculated with Olinda/EXM [16]. While patient-specific mass corrections were made to the Olinda S-values for the whole body, for other organs this was omitted because of insufficient anatomic information. The total absorbed doses to target regions were summed from the contributions of all source regions, also including contributions from daughter products of radium-223.

Clinical implications:

- WB doses delivered during the first administration were predictive of that delivered at the second.
- Urinary excretion was low, accounting for only 2 % of the total activity eliminated during the first 48 h following administration. Up to 34 % of the activity was eliminated in the faeces. By 6–8 days the total median excretion was 70 % (range: 9–78 %).
- The upper range of absorbed doses delivered to the red marrow and bone endosteum were higher than those based on the ICRP model (ICRP 67) [17]. The absorbed dose to the liver was found to be significantly lower than that previously expected [17].

Follow on work

Following the initial study additional processing of the dosimetry, biodistribution and pharmacokinetic data took place. This included further reporting on the excretion and WB retention up to 42 days following treatment [18]. These data demonstrated prolonged retention after 48 hours with an effective half-life of 8 days. Such retention indicated potential for further imaging after the 7 days of the original protocol, the feasibility of which was demonstrated by Pacilio et al in a separate Italian study [19]. The potential for predicting bone lesion response was reported in a post-hoc analysis using [^{18}F]Fluoride data acquired for five patients during the study [20]. In addition, a pharmacokinetic (PK) model was developed that proposed a multi-compartment bone model which matched well with individual biokinetic data of the study participants and proposed population rate constants [21]; example biological retention data from the model are shown (fig. y2).

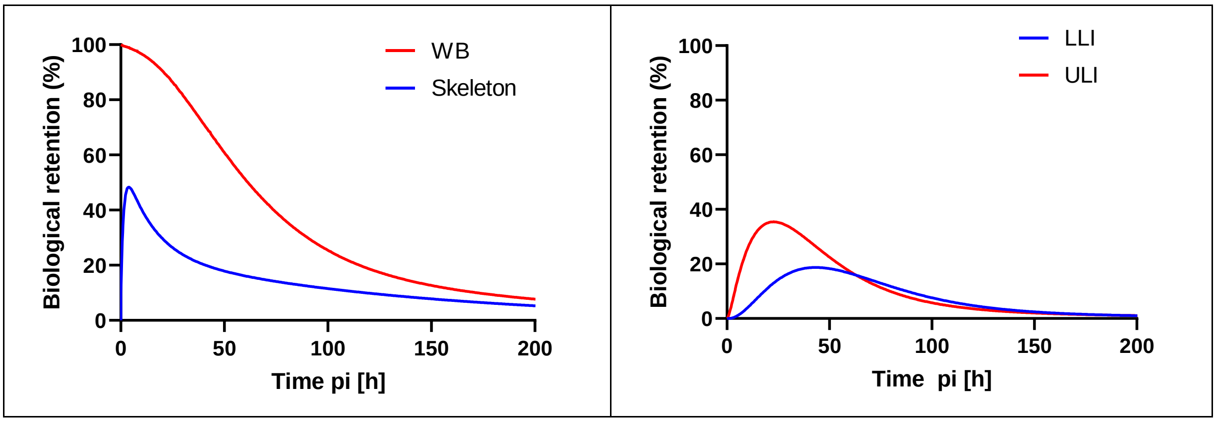

Figure y2: Biological retention data generated using the pharmacokinetic (PK) model of Taprogge et al. [21]. Data are shown for whole-body (WB), skeleton, lower (LLI) and upper large intestine (ULI).

[68Ga]Ga-NODAGA-RGDyK

Integrin-targeting radiopharmaceuticals have potential broad applications, spanning from cancer theranostic to cardiovascular diseases. The first human dosimetry of [^{68}Ga]Ga-NODAGA-RGDyK was conducted on five consecutive patients included in a clinical imaging protocol of carotid atherosclerotic plaques [22]. Five male patients underwent whole-body time-of-flight PET/CT scans 10, 60 and 120 min after tracer injection (200 MBq). Quantification of gallium-68 activity concentration was first validated in a phantom study. To be used as input in OLINDA/EXM, TACs were derived from manually drawn regions-of-interest over the following organs: brain, thyroid, lungs, heart, liver, spleen, stomach, kidneys, red marrow, pancreas, small intestine, colon, urinary bladder and whole body. Interestingly, the patients showed a prominent tracer concentration in the choroid plexuses, a cerebral structure not considered in standard source/target organ dosimetry models. Hence, a dedicated dosimetry analysis was performed for this anatomical structure. Because only male patients had been enrolled, female dosimetry was extrapolated from the male data. Effective doses were estimated according to both ICRP 60 and ICRP 103 assuming 30-min and 1-h voiding cycles.

<u>Set-up of the initial measurement protocol:</u>

- The administered activity of 200 MBq was set according to previous literature [23, 24], considered to provide sufficient image quality with a reasonably low effective dose exposure.
- Quantitative accuracy was assessed in a NEMA IEC PET Body phantom (NU 2) with sphere inserts, with the same acquisition and reconstruction parameters as used for patients. Accuracy on the recovery of the average activity concentration in regions without partial volume effects was within 6%.
- Small molecules are known to exhibit rapid uptake in tissues (within a few minutes), followed by rapid clearance from tissues (mainly driven by the physical decay of gallium-68 with a half-life of 68 min). Preclinical investigation with [^{68}Ga]Ga-NODAGA-RGDyK in mice [25] and previous in-human dosimetry studies with similar tracers labelled with gallium-68 and fluorine-18 [23, 24] confirmed the rapid elimination, driven mainly by the physical decay for most organs (using gallium-68, the effective half-lives were within 40-60 minutes, with the exception of the blood demonstrating faster biological clearance). Based on this information, three time points (allowing for mono exponential TIAC modelling) were considered in this study. PET acquisitions were performed at 10-, 60- and 120-minutes post administration, the latter corresponding to ~2-3 times the effective half-life for most source organs.

<u>Dosimetry evaluation:</u>

- Mono-exponential fit extended to infinity beyond the last measured data point was used to derive time-integrated activity coefficients for source organ TACs
- Absorbed dose evaluation for the red marrow VOIs was based on PET/CT data from the femoral head and the lumbar vertebrae L3–L4.

- Absorbed doses to target organs were derived with the OLINDA/EXM software, and both ICRP 60 and ICPR 103 tissue weighting factors were used for effective dose estimations (see fig. z1). The lack of tissue weighting factor for the choroid plexuses prevented this structure being explicitly included in the effective dose calculations.
- Specific absorbed dose assessment was performed for the choroid plexuses [26]. Accurate absorbed dose estimates in such an elongated structure, in the presence of relatively high-energy beta emission (typical of gallium-68 decay), necessitates specific modelling of a realistic geometry using Monte Carlo or ellipsoidal methods [26], as illustrated (fig. z2). Choroid delineation and mass estimation was based on gadolinium-enhanced axial T1-weighted images; organ total activity was assessed by PET, applying intensity threshold segmentation.

Clinical implications:

- Organs receiving the highest absorbed dose were the urinary bladder wall, the kidneys, and the small intestine (89.4, 46.3 and 30.1 μGy/MBq, respectively). Mean absorbed dose in the choroid plexuses was 39.6 μGy/MBq in the male cohort and 56.6 μGy/MBq for the female, from extrapolation.
- The 1-h voiding cycle extrapolation resulted in effective doses of 19.3 and 19.8 μSv/MBq according to ICRP 60 and ICRP 103, respectively. With a shorter urinary bladder voiding time of 30 minutes the effective dose reduces to 15.7 and 16.5 μSv/MBq, respectively.
- For possible future therapeutic applications, specific attention should be paid to delivered doses to the kidneys and potentially also to the choroid plexuses.

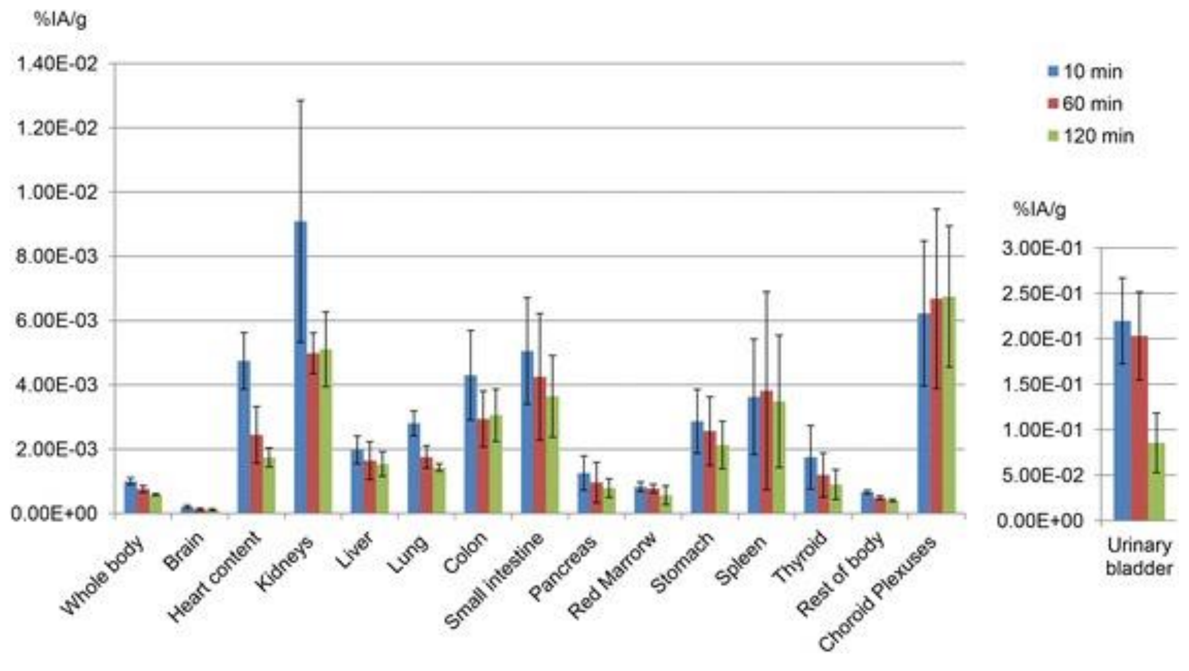

Figure z1. Organ activity corrected for gallium-68 physical decay. These data show the biological organ kinetics of ^{68}Ga]Ga-NODAGA-RGDyK during the observation time. Colour

bars represent the average percent of injected activity per gram of tissue (%IA/g) ± standard deviation. Reproduced with permission [22].

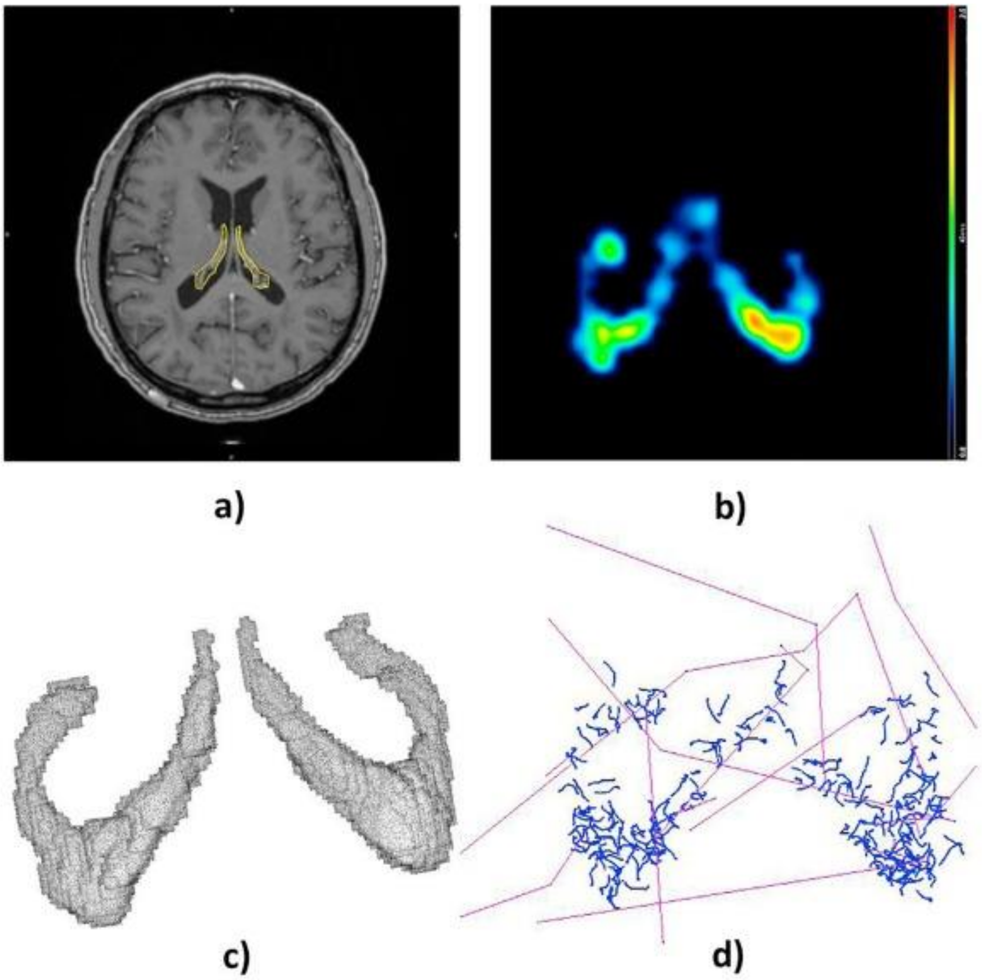

Figure z2. Monte Carlo modelling of absorbed dose deposition in the choroid plexuses. The panels show segmentation of the choroid plexuses superimposed on the T1-w MRI axial images (a), PET-based gallium-68 activity concentration (b), volume rendering (c), and simulated MC radiation propagation modelling (d). Reproduced with permission [26].

References

1. Kolstad, A., et al., *Phase 1/2a study of 177Lu-lilotomab satetraxetan in relapsed/refractory indolent non-Hodgkin lymphoma.* Blood Adv, 2020. **4**(17): p. 4091-4101.
2. Blakkisrud, J., et al., *Biodistribution and Dosimetry Results from a Phase 1 Trial of Therapy with the Antibody-Radionuclide Conjugate (177)Lu-Lilotomab Satetraxetan.* J Nucl Med, 2018. **59**(4): p. 704-710.
3. Blakkisrud, J., et al., *Red Marrow-Absorbed Dose for Non-Hodgkin Lymphoma Patients Treated with 177Lu-Lilotomab Satetraxetan, a Novel Anti-CD37 Antibody-Radionuclide Conjugate.* J Nucl Med, 2017. **58**(1): p. 55-61.
4. Blakkisrud, J., et al., *Tumor-Absorbed Dose for Non-Hodgkin Lymphoma Patients Treated with the Anti-CD37 Antibody Radionuclide Conjugate 177Lu-Lilotomab Satetraxetan.* J Nucl Med, 2017. **58**(1): p. 48-54.
5. Stokke, C., et al., *Pre-dosing with lilotomab prior to therapy with (177)Lu-lilotomab satetraxetan significantly increases the ratio of tumor to red marrow absorbed dose in non-Hodgkin lymphoma patients.* Eur J Nucl Med Mol Imaging, 2018. **45**(7): p. 1233-1241.
6. Blakkisrud, J., et al., *Myelosuppression in patients treated with (177)Lutetium-lilotomab satetraxetan can be predicted with absorbed dose to the red marrow as the only variable.* Acta Oncol, 2021. **60**(11): p. 1481-1488.
7. Londalen, A., et al., *FDG PET/CT parameters and correlations with tumor-absorbed doses in a phase 1 trial of (177)Lu-lilotomab satetraxetan for treatment of relapsed non-Hodgkin lymphoma.* Eur J Nucl Med Mol Imaging, 2021. **48**(6): p. 1902-1914.
8. Giesen, D., et al., *(89)Zr-PET imaging to predict tumor uptake of (177)Lu-NNV003 anti-CD37 radioimmunotherapy in mouse models of B cell lymphoma.* Sci Rep, 2022. **12**(1): p. 6286.
9. Maaland, A.F., et al., *Targeting B-cell malignancies with the beta-emitting anti-CD37 radioimmunoconjugate (177)Lu-NNV003.* Eur J Nucl Med Mol Imaging, 2019. **46**(11): p. 2311-2321.
10. Leggett, R.W., *A generic age-specific biokinetic model for calcium-like elements.* Radiat. Prot. Dosim, 1992. **41**: p. 183-198.
11. ICRP, *Age-dependent Doses to Members of the Public from Intake of Radionuclides - Part 2 Ingestion Dose Coefficients. ICRP Publication 67.* Ann ICRP, 1993. **23**(3-4): p. 1-167.
12. Henriksen, G., et al., *Significant antitumor effect from bone-seeking, alpha-particle-emitting (223)Ra demonstrated in an experimental skeletal metastases model.* Cancer Res, 2002. **62**(11): p. 3120-5.
13. Henriksen, G., et al., *Targeting of osseous sites with alpha-emitting 223Ra: comparison with the beta-emitter 89Sr in mice.* J Nucl Med, 2003. **44**(2): p. 252-9.
14. Nilsson, S., et al., *First clinical experience with alpha-emitting radium-223 in the treatment of skeletal metastases.* Clin Cancer Res, 2005. **11**(12): p. 4451-9.
15. Hindorf, C., et al., *Quantitative imaging of 223Ra-chloride (Alpharadin) for targeted alpha-emitting radionuclide therapy of bone metastases.* Nucl Med Commun, 2012. **33**(7): p. 726-32.
16. Chittenden, S.J., et al., *A Phase 1, Open-Label Study of the Biodistribution, Pharmacokinetics, and Dosimetry of 223Ra-Dichloride in Patients with Hormone-Refractory Prostate Cancer and Skeletal Metastases.* J Nucl Med, 2015. **56**(9): p. 1304-9.
17. Lassmann, M. and D. Nosske, *Dosimetry of 223Ra-chloride: dose to normal organs and tissues.* Eur J Nucl Med Mol Imaging, 2013. **40**(2): p. 207-12.
18. Pratt, B.E., et al., *Excretion and whole-body retention of radium-223 dichloride administered for the treatment of bone metastases from castration resistant prostate cancer.* Nucl Med Commun, 2018. **39**(2): p. 125-130.
19. Pacilio, M., et al., *The Italian multicentre dosimetric study for lesion dosimetry in (223)Ra therapy of bone metastases: Calibration protocol of gamma cameras and patient eligibility criteria.* Phys Med, 2016. **32**(12): p. 1731-1737.

20. Murray, I., et al., *The potential of (223)Ra and (18)F-fluoride imaging to predict bone lesion response to treatment with (223)Ra-dichloride in castration-resistant prostate cancer.* Eur J Nucl Med Mol Imaging, 2017. **44**(11): p. 1832-1844.
21. Taprogge, J., et al., *Compartmental Model for (223)Ra-Dichloride in Patients With Metastatic Bone Disease From Castration-Resistant Prostate Cancer.* Int J Radiat Oncol Biol Phys, 2019. **105**(4): p. 884-892.
22. Gnesin, S., et al., *First in-human radiation dosimetry of (68)Ga-NODAGA-RGDyK.* EJNMMI Res, 2017. **7**(1): p. 43.
23. Doss, M., et al., *Biodistribution and radiation dosimetry of the integrin marker 18F-RGD-K5 determined from whole-body PET/CT in monkeys and humans.* J Nucl Med, 2012. **53**(5): p. 787-95.
24. Kim, J.H., et al., *Whole-body distribution and radiation dosimetry of (68)Ga-NOTA-RGD, a positron emission tomography agent for angiogenesis imaging.* Cancer Biother Radiopharm, 2012. **27**(1): p. 65-71.
25. Buchegger, F., et al., *68Ga-NODAGA-RGDyK for alphavbeta3 integrin PET imaging. Preclinical investigation and dosimetry.* Nuklearmedizin, 2011. **50**(6): p. 225-33.
26. Amato, E., et al., *A Monte Carlo model for the internal dosimetry of choroid plexuses in nuclear medicine procedures.* Phys Med, 2018. **49**: p. 52-57.
